# Maxillary canines retained in the palate: tutorial for clinical cases

**DOI:** 10.1590/2177-6709.30.3.e25spe3

**Published:** 2025-10-13

**Authors:** Bruno Moreira das NEVES, David Silveira ALENCAR, Camila Silva Salgado dos REIS, Klaus Barretto dos Santos Lopes BATISTA, Jonas CAPELLI, Cátia Cardoso Abdo QUINTÃO, Felipe de Assis Ribeiro CARVALHO

**Affiliations:** 1 Universidade do Estado do Rio de Janeiro, Faculdade de Odontologia, Departamento de Odontologia Preventiva e Comunitária (Rio de Janeiro/RJ, Brazil).

**Keywords:** Impacted canines, Palatally displaced canines, Orthodontics, Interceptive treatment, Caninos impactados, Caninos deslocados palatinamente, Ortodontia, Tratamento interceptivo

## Abstract

**Introduction:**

The second most common form of dental impaction is the maxillary permanent canine, behind only the third molars. This condition affects approximately 2% of the population, with most cases (between 80% and 90%) resulting in impaction in the palate. The consequences of a canine impaction may include migration and root resorption in adjacent teeth, reduction of the dental arch perimeter, formation of cysts, and infections.

**Objective:**

We developed an interactive PowerPoint tutorial to assist professionals in selecting the most appropriate approach for cases of unerupted and palatally displaced permanent maxillary canines in young patients without or with minimal crowding in the maxillary arch.

**Conclusion:**

This tutorial is an auxiliary tool, and the treatment plan for this dental condition should be individualized to the characteristics of each patient.

## RESUMO

**Introdução:**

Os caninos superiores permanentes representam a segunda forma mais comum de impacção dentária, sendo superada apenas pelos terceiros molares. Essa condição afeta cerca de 2% da população e a maioria das ocorrências (entre 80% e 90% dos casos) resulta na impacção no palato. As sequelas de um canino impactado podem incluir migração e reabsorção radicular nos dentes adjacentes, redução do perímetro da arcada, formação de cistos e infecções.

**Objetivo:**

Com o propósito de auxiliar a abordagem mais adequada para casos de caninos superiores permanentes não irrompidos e palatinizados, em pacientes jovens, sem a presença de apinhamento ou com apinhamento leve na arcada superior, desenvolveu-se um tutorial interativo, em formato de apresentação do PowerPoint ^®^.

**Conclusão:**

Esse tutorial deve ser utilizado como ferramenta auxiliar, e o tratamento para essa condição dentária deve ser individualizado para as características de cada paciente.

## INTRODUCTION

The eruption of the maxillary canines is a complex and lengthy process, beginning from their point of formation (laterally to the pyriform fossa) until they are in functional occlusion. Due to the longer time to complete the eruption process, the canines are more susceptible to alterations in their eruption path, which may result in buccal or palatal eruption or impaction.^1^

The third molars are the most prevalent impacted teeth. The second is the permanent maxillary canines, with an estimated 2% of the population affected.^2^ ^,^ ^3^ Most retained canines are palatally displaced (80 to 90%), and around 10 to 20% are buccally displaced.^4^

In cases where the permanent maxillary canine erupts in an ectopic palatal path, extraction of the deciduous canine is the recommended treatment for patients between 10 and 13 years old.^5^ Before ten years old, this approach is not recommended, as spontaneous correction of canines may still occur,^6^ except for some very early somatic and dental development.

Some variables can be considered predictors for the success of correcting the eruptive path of retained permanent palatally displaced canines (PDCs). These include the extraction of the deciduous canine, the alpha angle (which represents the most horizontal or vertical inclination of the permanent canine about the midline), the sector measurement (which corresponds to the position of the crown of the permanent canine to the midline in the mesiodistal direction), and the patient’s age.^7^

When there is suspicion of PDCs, early diagnosis of this eruptive disorder confers an advantage upon the orthodontist, who can intervene by extracting deciduous canines or intercepting with the use of auxiliary appliances to obtain more space, and when the predictors for PDCs eruption added to a clinical unfavourable condition, a common approach is orthodontic traction, which is preceded by a stage of surgical exposure, which takes place using the open or closed technique,^8^ and auto transplantation of the PDCs can also be performed.^9^

Therefore, this article, associated with a tutorial, aims to use, based on scientific evidence, predictors of success for the orthodontic approach in patients with PDCs between the ages of 8 and 13, with the absence or presence of mild crowding in the maxillary arch, based on the analysis of clinical and radiographic characteristics of the retained tooth. This tutorial should be considered an auxiliary tool, and cases should be individualized when necessary.

## DIAGNOSIS OF PERMANENT CANINE RETAINED IN THE PALATE

The maxillary permanent canines generally erupt between 8 and 13 years old. Therefore, it is fundamental to carry out clinical and radiographic examinations to diagnose PDCs correctly. During the clinical examination, the professional can observe an altered eruption chronology, prolonged retention of the deciduous canine, and increased volume of the palatal mucosa can be observed.

Periapical radiographs, occlusal radiographs, and, in some cases, cone beam computed tomography (CBCT)^10^ are the most used radiographic examinations to determine whether the permanent canine is palatally displaced. Due to their simplicity and smaller radiation area, periapical radiographs are usually the first choice.

**Figure 1:**
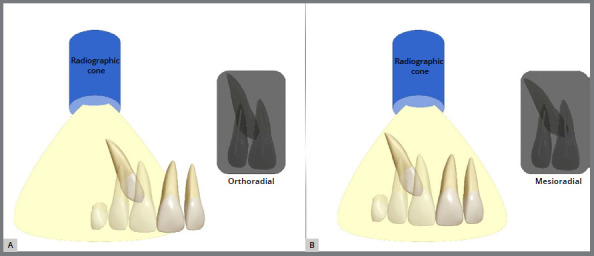
Clark’s root dissociation method to determine whether the permanently impacted canine is palatally or buccally displaced. Conventional orthoradial radiographic view **(A)** and mesioradial radiographic view with the canine moving mesially (overtaking the root canal of the central incisor) in the same direction of displacement of the radiographic cone, which indicates the canine retained by the palate **(B)**.

To ascertain whether the maxillary permanent canine is positioned buccally or palatally displaced, two periapical radiographs are taken to apply Clark’s root dissociation technique, which involves changing the horizontal angulation of the radiographic cone from the first to the second shot.^10^ Periapical radiography with an orthoradial incidence determines whether the canine is displaced palatally or buccally. When applying Clark’s technique, changing the position of the radiographic cone to a mesioradial position can state the displacement of the retained permanent canine palatally when the tooth moves in the same direction as the radiographic cone (mesial). If the canine moves distally, the displacement will occur for the buccal side (Fig 1).

## REQUIREMENTS TO USE THE TUTORIAL

This tutorial aims to reinforce, based on scientific evidence, the orthodontist’s possible decision-making regarding cases of maxillary permanent canines retained in the palate area.

For the best use of this tutorial, it will be necessary to have the following:

- Digital panoramic radiograph;
- ANB angle measure;
- Upper and lower intermolar distances measures;
- Intraoral clinical photographs.

## PREDICTORS OF SUCCESS FOR THE ERUPTION OF PALATALLY RETAINED PERMANENT CANINES

Studies indicate that the following factors predict the success in the spontaneous or after some intervention eruption in the PDCs: the alpha angle (α) value and the sector in which the PDCs are located, both obtained from panoramic radiographs.^5^ ^,^ ^7^ ^,^ ^11^

The deciduous canine extraction is the variable that most influence PDCs eruption, followed by angle α, sector, and patient age.^7^

Cases in which the PDCs tend towards a horizontal position (angle α ≥ 30º) represent a more unfavorable condition for spontaneous eruption. 

The sector in which the tooth is located is considered favourable when the PDCs have not exceeded the root of the lateral incisor towards the midline (sectors 1 and 2). 

The better is to evaluate as many predictors as possible together; however, if angle α and sector diverge, the value of angle α must be chosen.

## EVALUATION OF THE LONG AXIS OF THE PERMANENT CANINE (ALPHA ANGLE)

**Figure 2:**
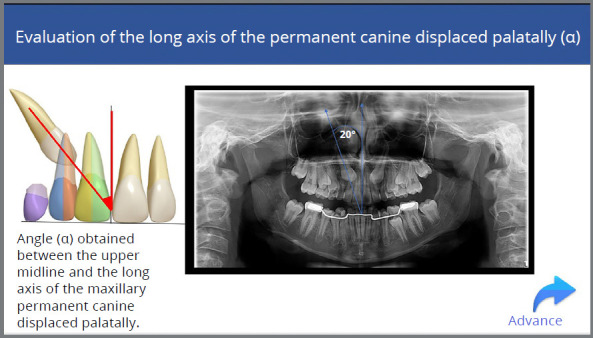
Illustration of the alpha angle and its marking on the long axis of the permanent right maxillary canine in panoramic radiography.

This assessment allows for observing the more vertical or horizontal position of the PDCs. This measurement is inferred through the angle α, obtained from the junction between the upper midline and the long axis of the maxillary permanent canine (Fig 2).^5^

In circumstances where the angle α is ≤ 20° and the PDCs are situated between sectors 1 and 2, it may be preferable to maintain the deciduous canine and make the clinical and radiographic follow-up that can be the most appropriate conduct for the spontaneous eruption of the PDCs.^7^

When the angle α presents its value between 21 and 29º, and the PDCs are located between sectors 1 and 3, extraction of the deciduous canine may be beneficial for the spontaneous eruption of the PDCs.^7^

When the angle α is ≥ 30°, and the PDCs are located between sectors 4 and 5, extraction of the deciduous canine is no longer beneficial for the eruption pattern of the PDCs, and immediate surgical exposure of the PDCs for orthodontic traction is indicated.^7^

## MESIODISTAL POSITIONING OF THE PDCS (SECTOR)

**Figure 3:**
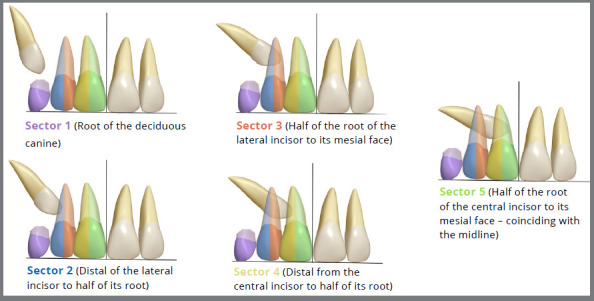
Illustration of the sectors, where in sectors 1 and 2, the canine does not surpass half of the lateral incisor root, and in sectors 3 to 5, the canine surpasses half of the lateral incisor root (Source: modified from Ericson and Kurol^5^, 1988).

The sectors are determined from 1 to 5, corresponding to the mesiodistal positioning of the PDCs crown about the lateral incisor, using the midline as a reference, mesiodistally (Fig. 3).^5^

In a classic study, Ericson and Kurol^5^ evaluated 46 PDCs, and radiographic examination revealed that 22 PDCs surpassed more than half of the root of the adjacent lateral incisor (sectors 3 and 4), and 14 (64%) PDCs exhibited standard eruption patterns after the extraction of the deciduous canine; in contrast, of the 24 PDCs that did not surpass half of the root of the adjacent lateral incisor before the extraction of the deciduous canine, 22 (91%) exhibited standard eruption patterns. 

Therefore, when evaluating the sector in which the PDCs are located, how much more the cusp tip extends beyond half of the root of the adjacent lateral incisor (towards the midline - sectors 3 to 5), the lower the probability of success in spontaneous eruption. The prognosis for successful eruption of the PDCs is more favourable when the cusp tip of the PDCs does not overtake half of the root of the adjacent lateral incisor, with a closer proximity to the deciduous canine (sectors 1 and 2) (Fig 3).

An interesting fact is that in the study by Ericson and Kurol^5^, no PDCs were identified in sector 5, which is considered to have the worst prognosis for eruption; in contrast, in the work of Naoumova and Kjellberg,^7^ in addition to not presenting PDCs in sector 5, they were also not identified in sector 1. Based on this information, the authors^7^ suggest that a PDC in Sector 1 would behave similarly to a PDC in Sector 2 and that a PDC in Sector 5 would act similarly to a PDC in Sector 4.^7^

## ANB ANGLE AND CLASS II RELATIONSHIP

Patients with maxillary PDCs may present with different types of skeletal classifications, as determined through cephalometric measurements, such as the ANB angle, which indicates the maxillomandibular relationship in the anteroposterior direction. According to the Tweed cephalometric analysis, an ANB angle value between 0° and 4.5° indicates a skeletal Class I pattern; on the other hand, a value greater than 4.5° indicates a skeletal Class II pattern, while a value less than 0° indicates a skeletal Class III pattern.^12^

When growing patients present Class II skeletal malocclusion, various treatment modalities are available, including extraoral appliances (EOA), rapid maxillary expansion (RME) combined with EOA, mandibular advancement appliances, one-phase treatment, etc. When coupled with the eruption of PDCs, these mechanics can improve spaces and anchorage. Without such intervention, there is an approximate 2.5 mm loss of anchorage of the maxillary first molars.^13^ Thus, these therapies may be employed when, in addition to PDCs, the patient presents clinical indications for molar distal movement or maxillary expansion associated with molar distal movement. These indications are necessary for specific approaches for PDCs, such as deciduous canine extraction.

## INTERMOLAR DISTANCE

Studies have demonstrated that in the presence of a transverse maxillomandibular skeletal discrepancy caused by maxillary constriction, performing an RME is an effective method of resolving transverse problems, thereby increasing the arch perimeter.^14^ ^-^ ^16^

In the study by Barros et al.,^17^ after RME, 70% of ectopic canines showed improvement in eruptive path. Although not a specific approach for PDCs, when associated with clinical indications for maxillary expansion, RME can improve the eruptive pattern of permanent canines.

Comparing success rates in PDCs eruption using RME followed by a transpalatal bar, Baccetti et al.^18^ demonstrated a success rate of approximately 80%, slightly higher than the 78% and 62% success rates observed in the studies by Ericson and Kurol^5^ and Power and Short,^19^ respectively, in which deciduous canine extraction was performed.

The RME can be an interesting alternative to improving the success rate in PDCs eruptions when transverse alteration justifies the indication for maxillary expansion, considering also the need to reduce the buccal corridor.

## DISTANCE BETWEEN PDCS AND THE OCCLUSAL PLANE (VERTICAL DISTANCE)

The vertical distance between the cusp tip of the PDC and the occlusal plane has been reported as a predictor for PDCs eruption in the work of Barros et al.,^17^ who determined a vertical classification relating the eruption level of the canine about the lateral incisor, where the canine may be at the level: 1) apical - cusp tip of the canine located at the apical or middle third of the root of the lateral incisor; and 2) occlusal - cusp tip of the canine located at the cervical third of the root or in the coronal region of the lateral incisor.

**Figure 4:**
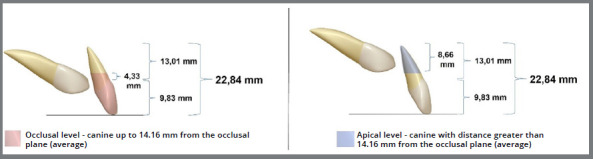
Adaptation of the vertical measurement performed by Barros et al.^17^ demonstrates the average occlusal and apical level values.

On average, a superior lateral incisor has a total length of 22.84 mm (9.83 mm corresponding to the crown and 13.01 mm corresponding to the root).^20^ Therefore, it can be assumed that each of the three-thirds of the root (apical, middle, and cervical) measures approximately 4.33 mm. With this information, it can be inferred that for the vertical distance to be used as a predictor variable for the eruption of the permanent canine and for it to be at the apical level (considered more unfavorable), it should be at a distance greater than 14.16 mm from the occlusal plane. On the other hand, to be at the occlusal level, the canine should be at a distance inferior to or equal to 14.16 mm from the occlusal plane (Fig 4). In the Ericson and Kurol^5^ study, the PDCs presented an average distance of 14.7 mm from the occlusal plane before treatment, classified at the apical level. After treatment, at six and 12 months of follow-up, all of them would be classified at the occlusal level, with averages of 11.7 mm and 9.2 mm, respectively, demonstrating improvement in the eruptive pattern after extraction of the deciduous canine, which is the main predictor for success in the eruption of the PDCs.

Some studies have reported vertical distance as a possible predictor of spontaneous eruption of PDCs. However, research has shown that this variable is not a reliable predictor of PDCs eruption.^5^ ^,^ ^7^ ^,^ ^19^ ^,^ ^21^ Due to the need for more standardization of samples across different studies regarding Nolla stages, this predictor may not be reliable; an 8-year-old patient would tend to have a higher canine with a less advanced Nolla stage than a 12-year-old patient.

Reducing the vertical distance from the PDCs to the occlusal plane is related to RME; thus, this variable does not apply to different intervention modalities that do not involve action in the maxillary transverse plane.^17^

## AGE AND NOLLA STAGE

This article provides a tutorial for patients between 8 and 13 years old without crowding or mild crowding based on studies that employed different approaches to improve the eruptive pattern of PDCs.^5^ ^,^ ^7^ ^,^ ^13^ ^,^ ^17^ In studies that included RME and EOA, the lowest mean age of patients was 8 years.^13^ ^,^ ^17^

The intervention involving deciduous canine extraction to promote improvement of the eruptive pattern of the PDCs should be considered in patients over ten years of age, as permanent canines may self-correct and erupt spontaneously up to this age.^6^

If PDCs are diagnosed late and the lateral incisor presents root resorption and the PDCs are too horizontal, other individualized approaches should be considered,^5^ being prudent, a request for cone beam computed tomography (CBCT) to evaluate possible damage to adjacent teeth better.

Regarding the Nolla stages,^22^ due to variations in the mean age of patients with PDCs, the study by Barros et al.,^17^ presented the lowest mean age of patients (8.43 years), with PDCs ranging between stages 7 and 8 (with at least 1/3 of the root formed or 2/3 formed). In the study by Naoumova and Kjellberg,^7^ the lowest mean age of patients was ten years, with PDCs ranging between stages 8 and 10 of Nolla (2/3 of the root form, root almost formed with open apex and root completely formed with closed apex, respectively). Based on these studies,^7^ ^,^ ^17^ we suggest that the permanent canines of the patients evaluated using this tutorial have at least 1/3 of the root formed, corresponding to stage 7 of Nolla.

## INDICATION OF CONE BEAM COMPUTED TOMOGRAPHY (CBCT)

The CBCT can be used for a variety of purposes in orthodontics, aiding diagnosis, and treatment planning in cases where three-dimensional evaluation of the position of impacted teeth and their relationship to adjacent teeth and neighbouring structures is required;^23^ assessment of the presence and severity of root resorption of teeth adjacent to retained canines;^24^ analysis of vestibular and lingual bone plates, and the remodelling effects that may result from tooth movement.^25^

Periapical radiography with the root dissociation method is indicated for the localization of impacted maxillary canines, premolars, and third molars. The Swiss Association of Dentomaxillofacial Radiology (SADMFR)^26^ guideline emphasizes that CBCT should be performed when there is information about pathological alterations or the possibility of surgical removal. An evaluation of the effective dose reduction between the ideal field of view (FOV) and the smaller FOV for impacted canines in CBCT scans, typically of 40∅ × 35 mm, showed a 33% reduction while holding other scan factors constant, suggesting that effective radiation doses received by young patients may be lower and safer.^27^

**Figure 5:**
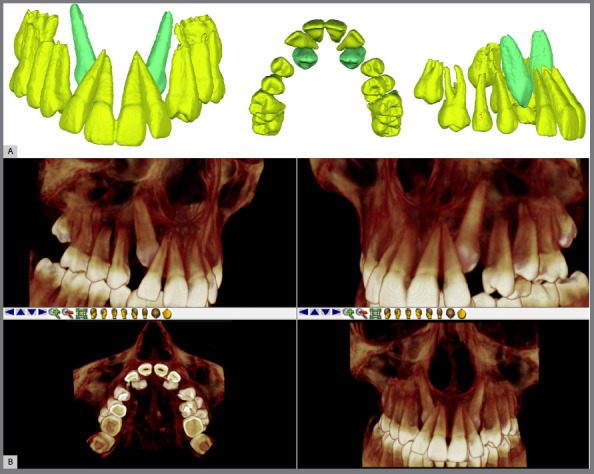
Three-dimensional segmentation demonstrating the contact relationship between the maxillary permanent canines (green) palatally located with the roots of the lateral incisors (yellow) **(A)**, performed through CBCT **(B)**.

Therefore, in cases where the PDC is in less favourable sectors and presents an angle α ≥ 30º with signs of intimate contact with adjacent roots (which may lead to root resorption), CBCT is suggested. Three-dimensional segmentation methods of CBCT allow decision-making regarding the most appropriate procedure (Fig. 5).

## SCIENTIFIC EVIDENCE

In medicine, guidelines guide clinical trials for specific pathologies or conditions. Systematic reviews based on randomized clinical trials should be consulted in cases of PDCs so that we can act based on the highest levels of evidence possible.

## FINAL CONSIDERATIONS

The purpose of this tutorial is to assist orthodontists and orthodontic postgraduate students in targeting approaches on patients aged 8-13 years with PDCs based on clinical and radiographic characteristics obtained from routine orthodontic documentation, such as panoramic and cephalometric radiographs, study model measurements, and intraoral photographs. The use of this tutorial, combined with the theoretical information contained in this article, may lead to CBCT scans being requested only in essential cases and may prevent undesirable effects that may result from non-intervention in PDCs at the appropriate time, such as resorption of adjacent teeth, reduction of arch perimeter, formation of cysts and infections, and aggravate of malocclusion.

## QR CODE AND LINK FOR ACCESS TO THE TUTORIAL IN POWERPOINT

[Fig f1a]

**Figure f1a:**
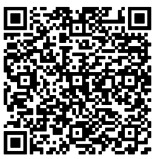
https://docs.google.com/presentation/d/1DGpCh1B1x__mnaRhT_PYRqJoWurGxdhW/edit?usp=sharing&ouid=105103570172128023942&rtpof=true&sd=true

## DEMONSTRATION OF HOW TO USE THE TUTORIAL

**Figure 6:**
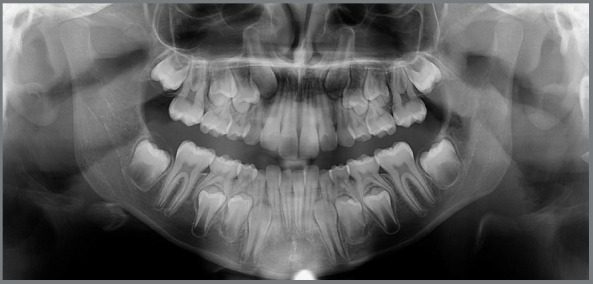
Panoramic radiograph taken at 8 years of age.

A female patient, aged 8 years, consulted an orthodontist, who requested a panoramic radiograph (Fig 6). The development of normal occlusion processes was observed, following the standard of normality. The permanent maxillary canines were at Nolla stage 8 and positioned higher, consistent with the patient’s age. Regarding predictors for successful eruption, the canines were located between sectors 1 and 2 with a more vertical inclination; characteristics that would favor an adequate eruptive pattern. Therefore, based on the observed characteristics, it was decided to proceed with clinical and radiographic follow-up.

**Figure 7:**
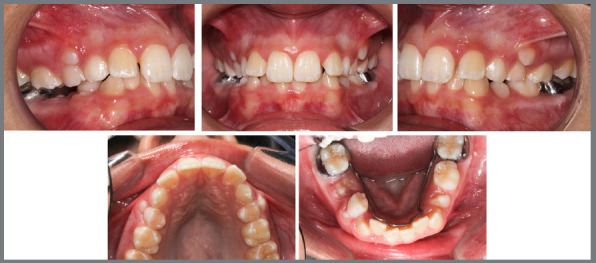
Initial intraoral photographs with cemented lingual arch.

Follow-up was conducted, and at a routine consultation at 10 years and 8 months of age, clinical examination revealed mixed dentition, Angle Class I relationship with a negative discrepancy in the lower arch, exaggerated overbite, maxillary lateral incisors projected in relation to the central incisors, and the tooth 45 initiating its eruption in the oral cavity. As a result, a lingual arch was fabricated and cemented to prevent mesial inclination of the 46 and to maintain the leeway space (Fig 7). Palpation indicated that the permanent maxillary canines were positioned in the palate. The upper and lower intermolar distances did not indicate a transverse problem (upper intermolar distance = 41 mm; lower intermolar distance = 37 mm); however, due to the presence of a wide buccal corridor, a strategy to reduce it would be considered during the planning.

**Figure 8:**
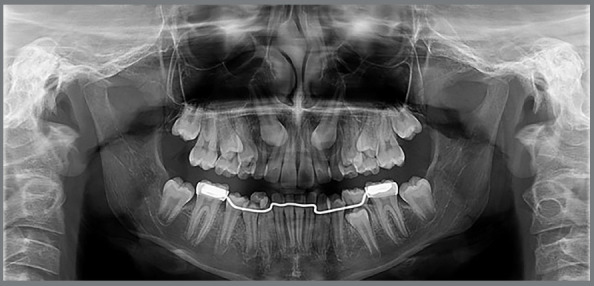
Initial panoramic radiograph with cemented lingual arch.

At this point, a new panoramic radiograph was requested (Fig 8).

**Figure 9:**
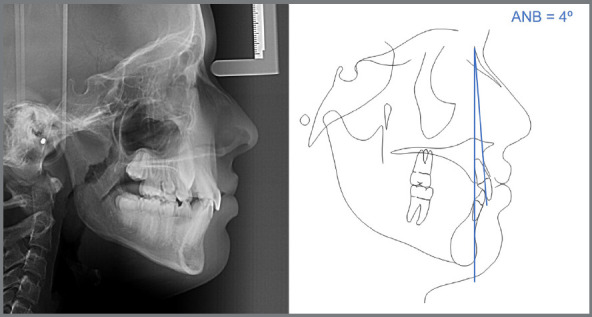
Initial radiograph and cephalometric tracing with measurement of the ANB angle.

In the analysis of the cephalometric radiograph, the patient presented a skeletal Class I relationship (ANB = 4º, SNA = 82º, SNB = 78º) (Fig 9). The panoramic radiograph showed the permanent maxillary canines in a more unfavorable mesiodistal positioning (sector) compared to the radiograph taken approximately two years prior (Fig 6), this indicated that, as they were extending beyond half the root of the lateral incisors, they were located between the sectors 3 and 4 (Figs 8 and 3). 

**Figure 10:**
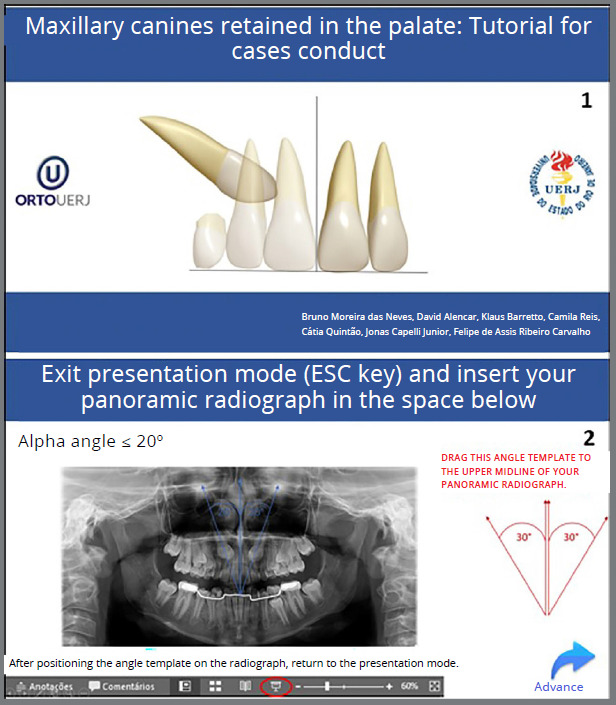
Use of the tutorial for case management. (1) Home page, (2) Alpha angle template positioned on the upper midline, demonstrating that the angle formed between the long axis of the permanent canines and the midline is ≤ 20º.

Given the following information: a) 10 years and 8 months, b) α angle ≤ 20º, c) permanent canines in sector 3, d) wide buccal corridor, e) ANB angle = 4º, f) Angle Class I with lateral incisors projected in relation to the central incisors; the tutorial was utilized to suggest the treatment approach, which was implemented, illustrating its correct indication and application (Figs 10 to [Fig f11] [Fig f12] 13, and 15).

**Figure 11: f11:**
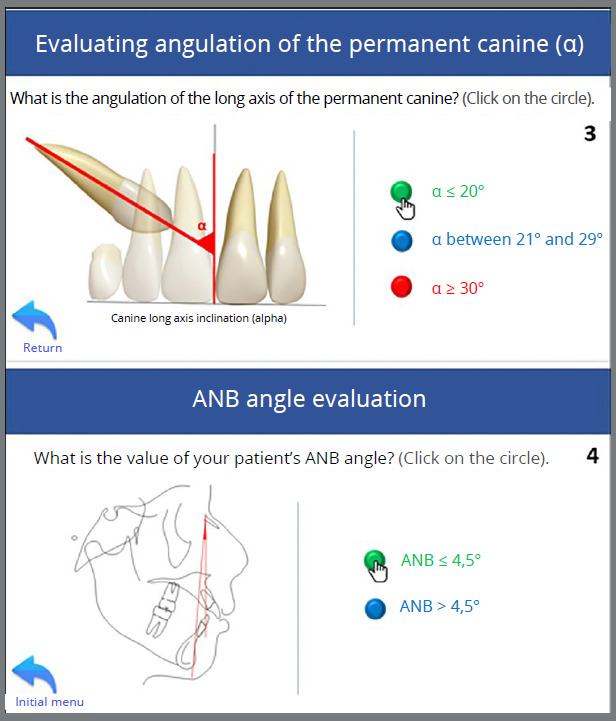
(3) Selection of the alpha angle value for the patient’s maxillary canines ≤ 20º. (4) Selection of the patient’s ANB angle recorded prior to using the tutorial (ANB = 4º).

**Figure 12: f12:**
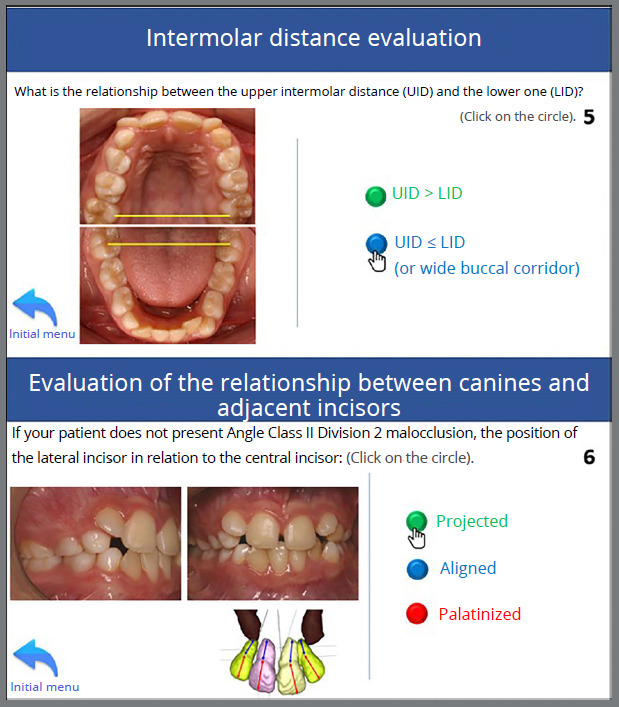
Selection of the relationship between the upper and lower intermolar distances of the patient, measured prior to using this tutorial - DIMS > DIMI, but with a wide buccal corridor and intent to reduce it during treatment (5). Selection of the inclination of the maxillary lateral incisor relative to the maxillary central incisor - in Class I, Class II division 1, or Class III patients, the corresponding option is marked; in Class II division 2, the lateral incisors are typically projected. The patient in this case presented projected lateral incisors relative to the central incisors (6).

**Figure 13:**
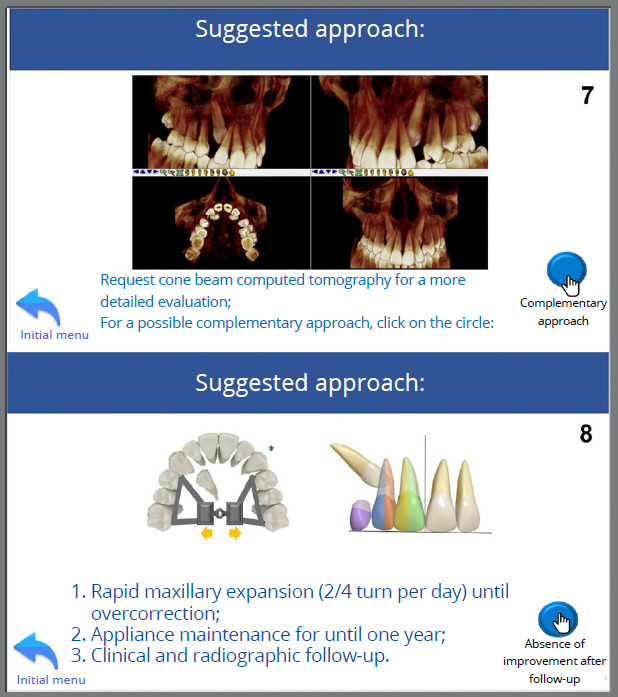
Due to the clinical characteristic of the lateral incisors projected in relation to the central incisors, which may indicate that the lateral incisors are being affected by the canines retained in the palate, the tutorial suggests requesting a CBCT of the region for a more thorough evaluation of the potential impacts of the canines on the roots of adjacent teeth (7); note that there is a blue circle indicating a complementary approach considering the predictive factors used in the case. Suggested approach after confirming through CBCT that the canines were not resorbing the roots of the lateral incisors, considering the predictors of the case in question (8); note that there is a blue circle indicating a new alternative in case of absence of improvement after treatment and follow-up.

**Figure 14:**
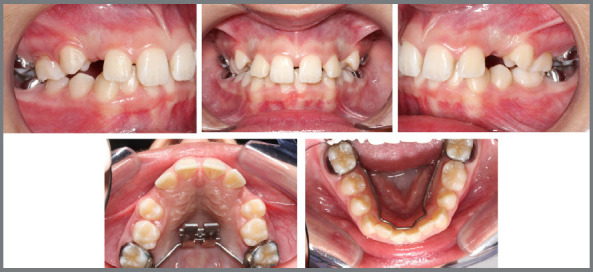
Intraoral photographs of the approach suggested by the tutorial, utilizing a maxillary expander and follow-up.

**Figure 15:**
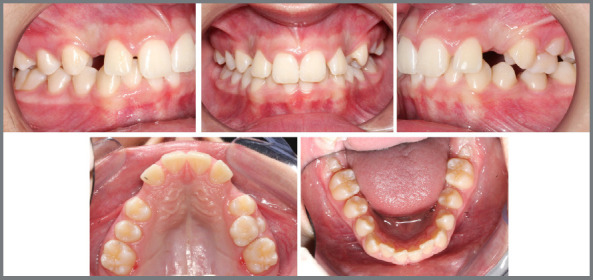
Intraoral photographs after the removal of the maxillary expander and the lingual arch.

As emphasized throughout the article, this tutorial serves as a guideline for management, which can be individualized as necessary. The orthodontist opted for the extraction of the maxillary deciduous canines. Thus, even with the extraction of the deciduous canines, the suggested approach was to request a CBCT, which, through 3D segmentation, confirmed that the permanent canines were not causing damage to the lateral incisors (Fig 5). Following the steps of the tutorial and selecting the complementary approach (Fig 13), a new treatment approach including maxillary expansion and clinical and radiographic follow-up was proposed (Fig 13).

Following the guidelines of the tutorial, a Hyrax appliance was fabricated and installed, adhering to the protocols for activation, stabilization, and monitoring over a period of 6 months (Fig 14).

The lingual arch and the Hyrax appliance were removed simultaneously, allowing for the observation of the planned reduction of the buccal corridor prior to the initiation of treatment, as well as the complete eruption of the mandibular second premolars without mesial inclination of the first permanent molars (Fig 15).

**Figure 16:**
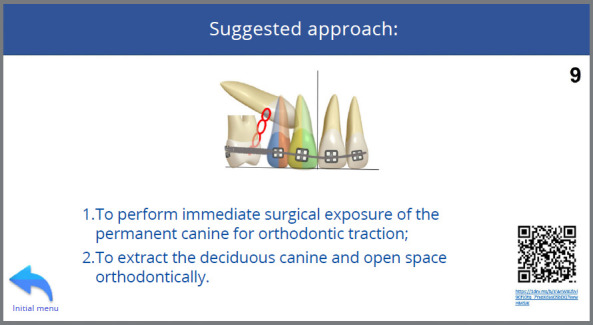
Following the absence of significant improvement with the previously suggested approach, a new approach involving surgical exposure for orthodontic traction was suggested by the tutorial (9).

After expansion and stabilization, the permanent canines had not erupted, and the tutorial was accessed again by selecting the option ‘absence of improvement after follow-up’ (Fig 13), which redirected to a new approach (Fig 16).

The new approach involved the extraction of the deciduous canines and surgical exposure of the permanent canines for orthodontic traction. However, since the deciduous canines had already been extracted, the orthodontist’s planning continued to follow the tutorial’s recommendations, with the surgical exposure of the permanent canines for orthodontic traction (Fig 16).

**Figure 17:**
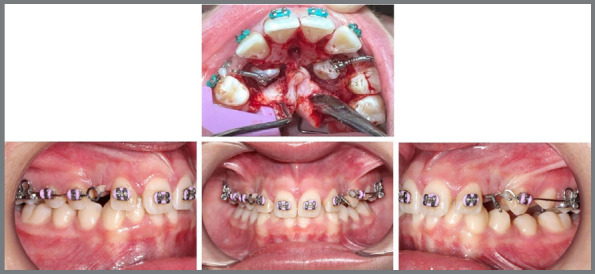
Surgical exposure of the impacted canines with the bonding of accessories connected to nickel-titanium springs; and intraoral photographs showing the activated springs tied to the helicoids of the segmented archwires for the traction mechanics of the canines.

The surgical exposure of the impacted canines was performed to bond accessories connected to nickel-titanium springs to the crowns of the impacted canines (Fig 17). These springs were activated and tied to the helicoids of .019 x .025-in segmented stainless steel archwires (Morelli, São Paulo, SP, Brazil). The archwires were then inserted into the fixed appliance to enhance the anchorage for the traction of the impacted canines (Fig 17).

**Figure 18:**
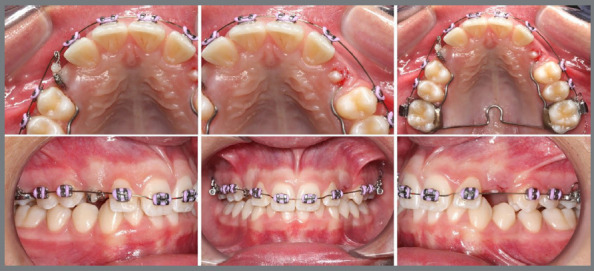
Mechanics for traction of the PDCs after 3 months, with a modified transpalatal bar showing improvement in the position of the permanent canines; with a closed nickel-titanium spring connected to the accessory bonded to the right canine, while the left canine had already erupted into the oral cavity, eliminating the need for maintenance of the nickel-titanium spring.

To counteract the adverse effects of the vestibular movement vector of the canines, which tends to displace palatally the premolars and constrict the arch, a modified transpalatal bar made of 0.9 mm stainless steel wire was employed. This bar contours the palatal surfaces of the premolars and extends to the mesial surface of teeth 14 and 24 (Fig 18).

**Figure 19:**
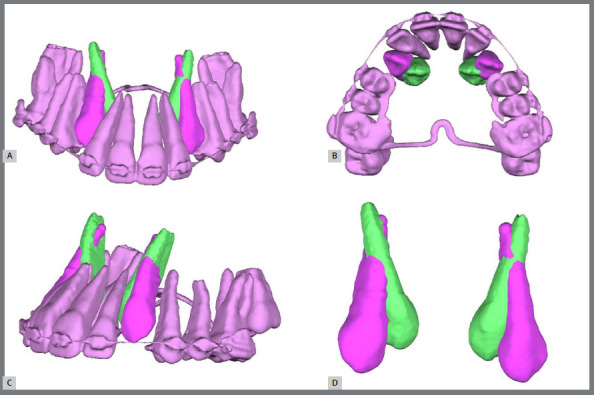
3D superimposition of the segmentations of the canines before (green) and after (purple) the traction mechanics, demonstrating improvement in the angulation and position of the impacted canines in the frontal view **(A)**, occlusal view **(B)**, left lateral view **(C)**, and isolated superimposition of the canines **(D)**.

The upper appliance was installed through the direct bonding of metallic brackets (Alexander prescription .022” x .028”) from teeth 15 to 24. After three months of activating the springs for traction, it became possible to include tooth 25 and continue with alignment and leveling (Fig 18). At this point, a second CBCT was performed to evaluate the new position of the permanent maxillary canines and the roots of the lateral incisors following the interventions. A 3D superimposition demonstrated an improvement in the angulation of the canines, absence of resorption in the lateral incisors, and successful traction (Fig 19).

**Figure 20:**
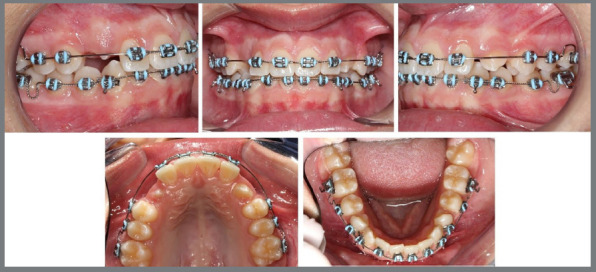
Intraoral photographs of the maxillary canines in a more suitable position in the arch (particularly the left canine), and lower fixed orthodontic appliance installed for the continuation of orthodontic treatment.

Five months after the start of the orthodontic traction mechanics, the permanent maxillary canines were better positioned in the arch; and the orthodontic treatment continued with fixed appliances, without including the canines at this time. The sequence of alignment and leveling was followed, a and a tietogether of the maxillary incisors was performed to preserve the space for the canines (Fig 20).

**Figure 21:**
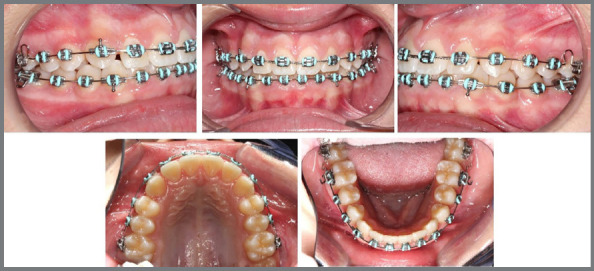
Intraoral photographs with complete fixed orthodontic appliance, with maxillary canines included and being aligned and leveled.

In the course of treatment, with the maxillary canines in a favorable position for bonding after 3 months, the complete assembly of the appliance was performed, the orthodontic mechanics are being continued with better-positioned canines, two months after bonding (Fig 21).
